# Is there a difference between heat-capsaicin induced low back pain and placebo for neural oscillations and inflammatory blood markers? An experimental randomized crossover study

**DOI:** 10.3389/fpain.2025.1621810

**Published:** 2025-09-11

**Authors:** Mona Frey, Allyson Summers, Sarah D. Power, Felipe C. K. Duarte, Diana E. De Carvalho

**Affiliations:** ^1^ Faculty of Medicine, Memorial University of Newfoundland and Labrador, St. John’s, NL, Canada; ^2^ Faculty of Engineering and Applied Science, Memorial University of Newfoundland and Labrador, St. John’s, NL, Canada; ^3^ Division of Research and Innovation, Canadian Memorial Chiropractic College, Toronto, ON, Canada

**Keywords:** experimental low back pain, heat-capsaicin, electroencephaloagraphy (EEG), cytokines, placebo, brainwaves, EEG band power

## Abstract

**Purpose:**

Low back pain is difficult to study due to its heterogeneity. Inducing back pain experimentally, with an established model such as heat-capsaicin, would beneficially control for some variability. How heat-capsaicin affects neurophysiological factors relevant to back pain is currently unknown, therefore, this study used a randomized crossover design with the aim to explore the differences between heat-capsaicin and placebo on brain activity and blood markers.

**Methods:**

18 healthy participants completed two sessions: heat-capsaicin (45°C heat + capsaicin) and placebo (reduced heat + placebo). Pre- and post-pain-induction/placebo, electroencephalogram and blood draws were taken, and perceived pain was rated with a 100 m visual analog scale. Band power was calculated for theta (4–8 Hz), alpha (8–13 Hz), beta (13–30 Hz), gamma1 (30–58 Hz), and gamma2 (62–100 Hz) for six brain regions. An immune assay was run on plasma in duplicate for cytokines IL-1β, IL-6, IL-10, and TNFα. A repeated measures ANCOVA was run for all variables comparing between conditions (heat-capsaicin, placebo) with baseline measures as covariates. A Pearson's correlation was used to determine the relationship between perceived pain ratings and brain wave and blood biomarkers.

**Results:**

The heat-capsaicin model induced transient mild to moderate pain which was significantly higher than placebo (24.50 vs. 0.39; *p* < 0.001). Brain wave and blood biomarkers were not significantly different between heat-capsaicin and placebo (*p* ≥ 0.05) or correlated to perceived pain ratings (*p* ≥ 0.15).

**Conclusion:**

Levels of perceived pain did not relate to neurophysiological changes that may occur immediately after heat-capsaicin exposure. Although changes have been found with other pain models and clinical low back pain, a statistically significant systematic response was not measurable using blood cytokine markers immediately after pain induction and may take longer to develop.

## Introduction

Low back pain (LBP) is the leading cause of years lived with disability (1). About 90% of cases are considered non-specific LBP (2), resulting in a heterogenous condition that is difficult to study in a controlled manner. Experimental induction of pain allows for within-subject comparisons, control between-subject variability, and increase statistical power. It can produce an acute pain response without having to consider confounding factors which makes experimental pain ideal to investigate the mechanisms that result in the adaptations of movement and function observed with clinical LBP. Two pain models are typically used to induce experimental spinal pain: hypertonic saline injections and the heat-capsaicin (HC) model (3–5). The HC model is less invasive and thus more practical: it is applied topically and can induce stable and long-lasting pain with minimal risk of tissue injury (6, 7).

Applied over the low back, previous studies have found that 15–20 min of HC can induce changes in biomechanical parameters typically associated with chronic LBP such as impaired local dynamic stability and muscular contributions to lumbar spine rotational stiffness (5). Similarly, HC applied to the cervical spine has been shown to alter stability within the cervical and thoracic spine and resulted in a tightening postural strategy (3, 4). Showing that these changes also occur in response to experimental pain, suggests that the adaptations are not due to long-term changes or confounders but rather related to short-term changes related to the acute pain response. However, the specific mechanism resulting in these adaptations remains unclear. Physiologically, the HC model evokes central sensitization and its symptoms of hyperalgesia and allodynia which are also proposed to be a mechanism involved in chronic LBP, but it is unclear how similar the HC pain is to clinical LBP.

Systemic inflammation and neural oscillations have shown to be altered with clinical LBP (8, 9) and could potentially contribute to the adaptations of biomechanical parameters found in recent studies. While neither have been explored yet with HC protocols to induce LBP, both have been investigated with experimental pain. An increase in inflammatory cytokine IL-6 has been found 30–120 min after HC pain induction over the calf but was not measured within the 15–20 min timeframe that most biomechanics studies use. Similarly, studies show activity altered brain wave activity in response to induced pain with changes depending on the pain model, intensities and durations (10, 11). Most consistently, alterations in the gamma bands have been shown (11). The response to pain should be understood as a complex spectral-temporal-spatial response where phasic pain has been associated with changes over the sensorimotor cortex while longer periods of pain (10 min) have been positively associated with increased gamma power over the medial prefrontal cortex (12).

Since the HC model is commonly used to induce experimental LBP, it is important to determine what neurophysiological changes occur to determine how well it models clinical LBP. Thus, the purpose of this study was to investigate if differences in blood and brain biomarkers occur between experimental LBP and placebo. A secondary objective was to determine if perceived pain ratings correlate with these objective physiological measures. We hypothesized that blood and brain biomarkers were different with HC pain compared to the placebo condition. We further hypothesized that there was a moderate correlation between biomarkers and pain ratings.

## Materials and methods

This study is an experimental randomized crossover design and has been reported according to the CONSORT extension for randomized crossover trials (13). The study was not registered.

### Participants

Convenience sampling was used for this study with a target size of 15 due to limited resources/funding. As 3 participants did not rate any pain in response to the HC pain model, we collected 18 participants but decided not to exclude the non-pain developers. Thus, 18 pain-free, healthy adults were recruited from the local community through poster, email, and social media advertisements. Participants were pre-screened and excluded if they had diabetes, high blood pressure or inflammatory disease, severe chronic pain (>4 pain days/month for at least three months), neurological disease, disorder or injury, psychiatric disorder, cognitive impairment, pregnancy, or communicable disease (i.e., Hepatitis C, HIV), or if they had a perceived pain rating greater than 10 mm on a 100 mm digital VAS at the beginning of the sessions. Participants were asked to refrain from engaging in strenuous exercise and from taking pain or anti-inflammatory medication (i.e., ibuprofen or acetaminophen) for 24 h prior to the data collection sessions. They were asked to refrain from smoking and ingesting caffeine or alcohol for 8 h prior to each session. Additionally, sessions were scheduled at least 48 h apart to prevent carry-over effects. The study received ethical approval from the provincial Health Research Ethics Board (#HREB 2220467) and all participants provided written informed consent prior to participating.

### Data collection

**Figure 1:**
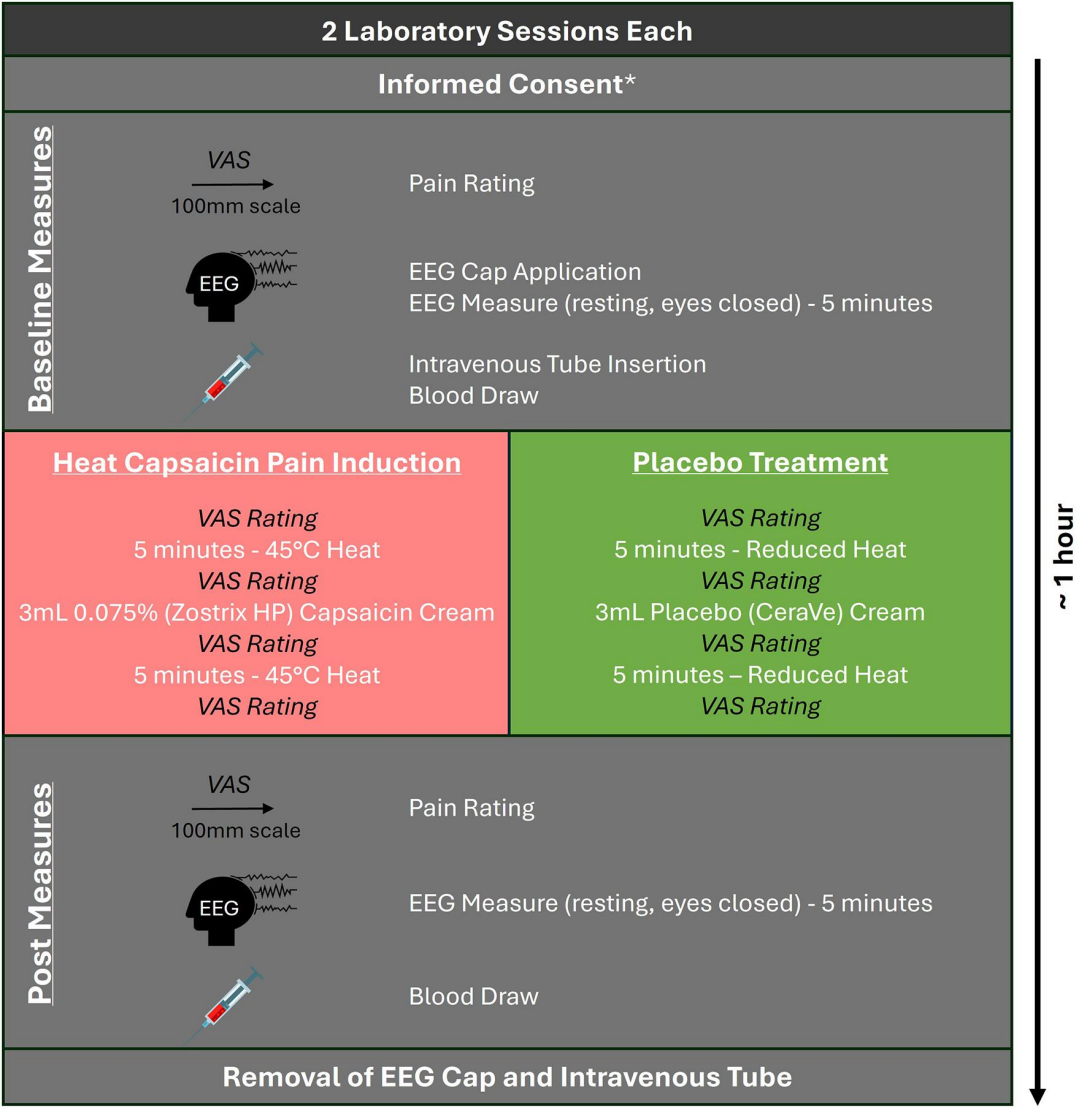
Data collection protocol. *Informed consent was only collected prior to the first session.

All participants completed two laboratory sessions which took approximately one hour each. Data was collected between January and April 2023. Both sessions were identical but used the HC model for one and a placebo treatment for the other in a block randomized order (Figure 1). The sequence of block randomization was determined prior to the first data collection. A researcher external to the study generated the randomization scheme (Microsoft Office, Excel v16) and prepared a set of opaque envelopes to be used by the research team. Prior to each data collection, a research assistant who only minimally interacted with the participant, handled the envelope and set the heating pad and laid out the cream used for the HC and placebo conditions. The main researcher who was the main contact for the participants and the participants were blinded.

Prior to the first session, participants completed an informed consent form and a screening questionnaire to ensure eligibility based on the exclusion criteria. At the beginning of each session, participants then completed a baseline pain rating on a digital 100 mm visual analog scale (VAS). Participants were equipped with an EEG cap and a 5-minute baseline trial was collected. For all EEG trials, the lights were turned off and participants were seated and instructed to close their eyes, remain still, and try to not think of anything. Next, an intravenous tube was applied to participants' cubital vein for the blood draws, and the baseline blood sample was taken. Following the baseline measures, the placebo or HC condition was induced. For both, an electric heating pad (TheraTherm) set to 45°C was placed over the low back and held in place with a low back support belt. For the placebo condition, the heating pad was turned off upon application. For the HC condition, the temperature was maintained at 45°C. The length of the support belt was measured to ensure even tension between the two sessions. The heating pad was removed after 5 min to apply 3 ml of cream (Zostrix HP 0.075% capsaicin or placebo—CeraVe Moisturizing Cream) within a 5 × 15 cm rectangle centered between the second and fourth lumbar vertebra on the participant's back. Both the participant and the researcher conducting the experiment were blinded to the condition, with an assistant setting the heating pad temperature and preparing the cream. The area where the cream was applied was then overlaid with plastic wrap and the heating pad was reapplied to the low back with the belt. After 5 minutes, a second 5-minute EEG trial was collected, followed by the second blood draw. Along with the baseline rating, participants rated their pain on the 100 mm VAS at three other points during the session: immediately prior and after the second EEG trial and immediately after the second blood draw. Finally, all equipment was removed, and the cream was wiped off with a wet, cold towel.

### Instrumentation & data analysis

#### Visual analogue scale (VAS)

Participants rated their perceived pain throughout the study via a digital 100 mm VAS presented on a tablet (Pain Rating Scales v.2.1, ETZ.soft). The scale was anchored at “0 mm = no pain” and “100 mm = worst pain imaginable”. Participants used their finger to set the location of the bar corresponding to their perceived level of pain at that time. The VAS score was defined as the rated score (distance from 0 in mm) for each time point. The first VAS rating was used as the baseline and the highest VAS rating post-pain/placebo induction was used as the post-condition value.

#### Electroencephalography (EEG)

A 32-channel electrode system (ActiChamp, Brain Products GmbH, Gilching, Germany) was used to measure brain activity. Electrodes were placed based on the 10–20 international system and FCz was used as the reference electrode. Electrode impedance was kept below 10 kΩ to ensure signal quality, and data was collected at a sampling frequency of 500 Hz.

EEG data were analyzed in MATLAB (R2021a) using the EEGLab Toolbox and custom code. First, a 0.1 Hz high-pass FIR filter was applied to each EEG trial, and each electrode channel was re-referenced to the average of all electrodes. Starting from the end of each trial working backwards, the first 60 s of artefact-free data were extracted for further analysis. Channels were visually inspected for noise and excluded if noise was detected throughout the full 5-minute trial. Following, the baseline was removed by subtracting the average of the 60 s of artefact-free data. To reduce the number of comparisons in the statistical analyses, EEG channels were grouped into six regions: frontal (Fp1, Fp2, F7, F3, Fz, F4, F8), central (FC5, FC1, FCz, FC2, FC6, C3, Cz, C4), temporal (FT9, FT10, T7, T8, TP9, TP10), parietal (CP5, CP1, CP2, CP6, P7, P3, Pz, P4, P8), occipital (O1, Oz, O2), and global brain (all electrodes) (14). Then, the average absolute power in five different frequency bands was calculated for each EEG region using the MATLAB function “bandpower”. Frequency bands were defined as: Theta 4–8 Hz, Alpha 8–12 Hz, Beta 13–30, Gamma1 30–58, Gamma2 62–100 (15). Absolute frequency band power was chosen since our within-subject, counterbalanced design and use of baseline EEG as a covariate in the ANCOVA allowed us to account for individual and session-level variability in signal amplitude. All analyses were conducted blinded, and trials were sorted into the placebo and HC conditions post-analysis.

#### Blood draws & markers of inflammation

Blood draws were collected by a registered nurse using an intravenous catheter (BX/50 Insyte Vialon Peripheral Venous IV Catheter, 24 g X0.75” Yellow., Life Supply., Surrey, BC, Canada) that was placed in the participants' cubital vein prior to the first blood draw and was kept in until the end of the data collection. 5 ml of blood were extracted into an EDTA anti-coagulant vacutainer coagulant (Vacutainers 6 ml Lavender, Life Supply., Surrey, BC, Canada). Prior to drawing the second blood sample, the vein was flushed with saline.

Upon data collection, blood samples were centrifuged for 15 min (4 °C, 3,000 rpm). The EDTA plasma was then stored in 0.6 ml MCTs (Low-Retention 0.6 ml MCT Snap Tops, Fisher Scientific Company., Ottawa, ON, Canada) and stored at −80°C. After all participants were collected, the samples were shipped to Eve Technologies Corporation in Calgary, Alberta where a human high sensitivity bead-based custom multiplex assay for interleukin 10 (IL-10), interleukin-1β (IL-1β), interleukin-6 (IL-6) and tumor necrosis factor alpha (TNF-α) was conducted in duplicate. These cytokines were chosen due to their suggested role underlying the pathophysiology of LBP (9, 16). Cytokine concentrations were determined in picograms per millilitre (pg/ml). Eve Technologies Corporation received all samples without indicating their experimental condition. Samples were organized into HC and placebo conditions post-analysis.

### Statistical analysis

All eighteen participants were included in the analyses. Averages, standard deviations, and 95% confidence intervals were calculated for all outcomes. Post-condition measures were statistically evaluated using repeated measures analyses of covariance based on linear mixed effects models that considered a fixed effect of condition (HC pain vs. placebo) and random intercepts for each participant. The pre-condition measures were entered into the models as the covariates. Paired comparisons between the two levels of condition with Tukey adjustments for multiple comparisons were conducted as *post-hoc* statistical tests. All statistical procedures were performed in R [version 4.4.1 (17)] and used the lme4 (18), car (19) and emmeans (20) packages. To determine whether perceived pain ratings correlate with the objective markers, a Pearson's correlation analysis was run between the change in VAS scores and change in EEG and blood measures including both placebo and pain using SPSS (version 27, IBM, Armonk, NY, USA). Change scores were calculated by subtracting the pre-condition values from post-condition values. Outcomes considered in both the ANCOVA and correlation analyses included: peak VAS scores, blood marker concentrations (TNF-α, IL-1β, IL-6, IL-10), and average absolute EEG frequency band power (theta, alpha, beta, gamma1, and gamma2 bands for the frontal, central, temporal, parietal, occipital, and global region respectively). The level of significance was set at *p* ≤ 0.05.

## Results

**Figure 2:**
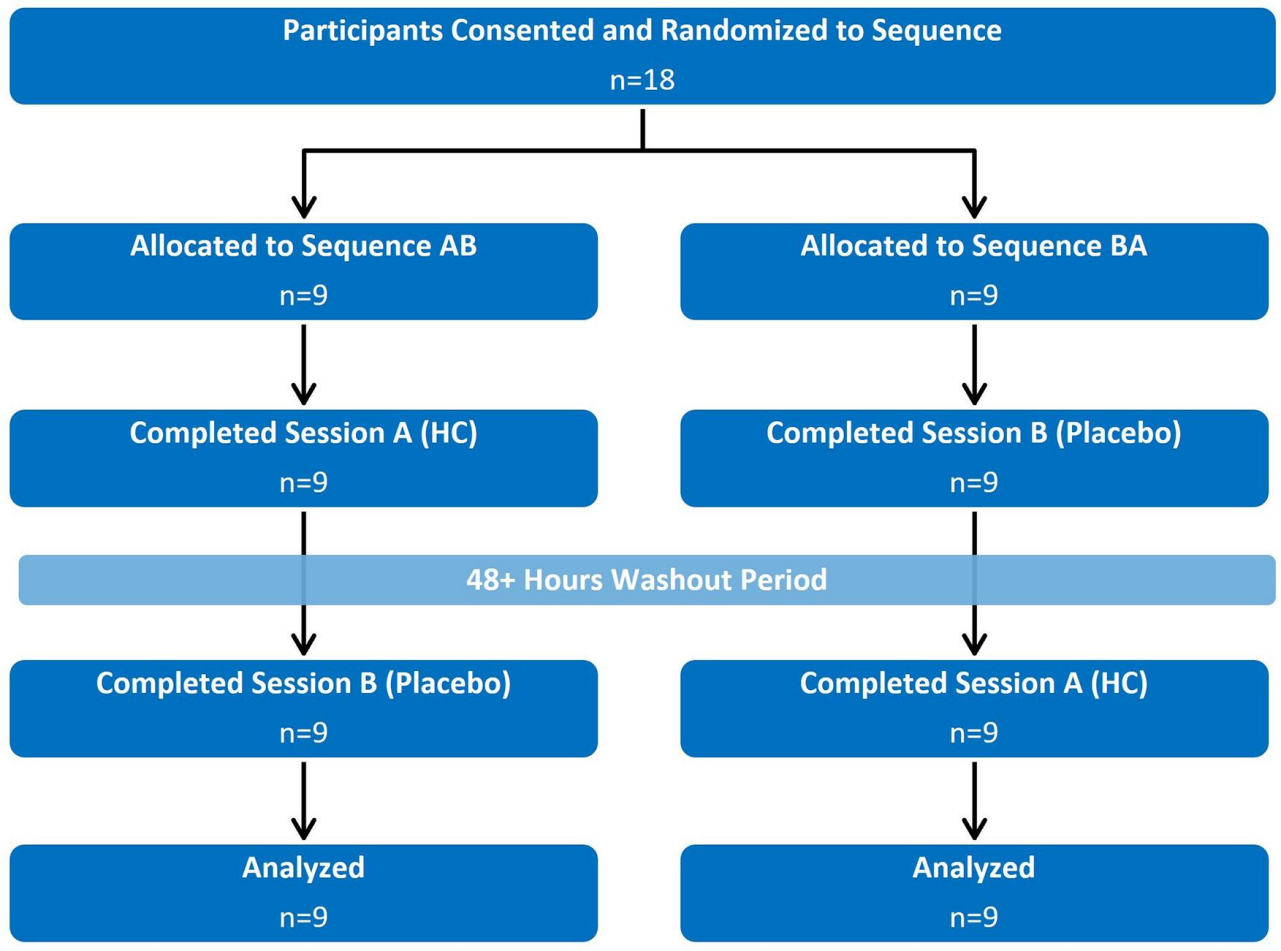
Flow diagram demonstrating the randomized crossover design. Participants were pre-screened for inclusion/exclusion criteria prior to consenting and randomization.

Eighteen participants (9 males, 9 females) were recruited and completed both sessions (Figure 2). Participant characteristics are reported in Table 1. The two sessions were on average 9.72 ± 10.94 days apart.

**Table 1:** Average participant characteristics (and standard deviations) by sequence and condition (no participants dropped out).

Outcomes	Sequence		Condition	
	AB:	BA:	Placebo	HC Pain
	1. HC	1. Placebo		
	2. Placebo	2. HC		
Number of Participants	9	9	18	18
Sex	5M, 4F	4M, 5F	9M, 9F	9M, 9F
Age (years)	31.13 (9.47)	28.40 (12.15)	29.61 (12.34)	29.61 (12.34)
Height (cm)	178.63 (7.24)	176.78 (12.42)	182.75 (12.04)	182.75 (12.04)
Mass (kg)	81.13 (9.81)	79.11 (10.98)	80.06 (11.79)	80.06 (11.79)

**Figure 3:**
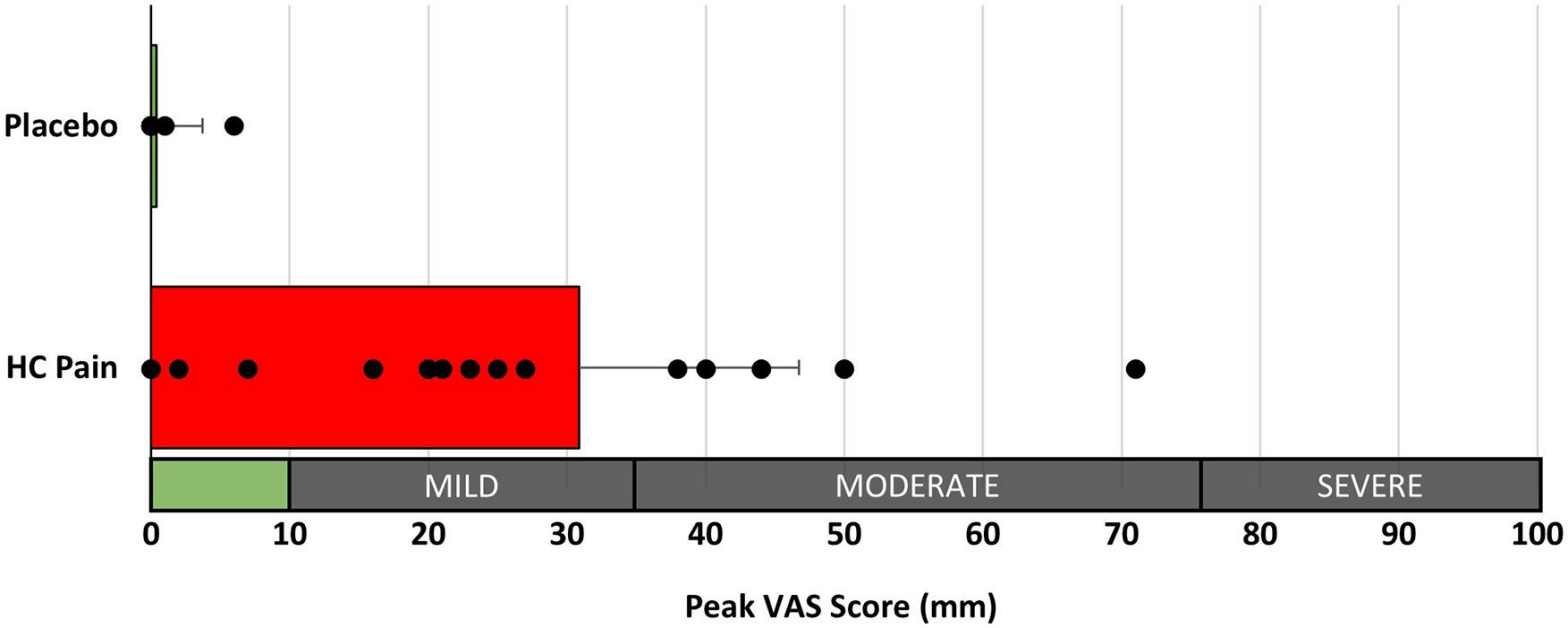
Peak perceived pain ratings (100 mm VAS) were significantly different between the placebo and HC pain conditions (*p* < 0.001). Interpretation of pain scores is given along the x-axis.

The HC pain model induced transient mild to moderate pain in 14 of the 18 participants which was significantly higher than the placebo condition (*p* < 0.001; Figure 3, Table 2). No adverse events were reported.

**Table 2:** Average outcome measures (and standard deviations) pre- and post-condition for both placebo and HC pain. *p*-values are given for the repeated measures ANCOVA, and r- and *p*-values for the Pearson Correlation analysis, between the change in pain ratings and changes in each neurophysiological outcome measure.

Outcomes			Pre-condition		Post-condition		ANCOVA	Pearson’s correlation	
			Placebo	HC pain	Placebo	HC pain	*p*	r	*p*
Peak Pain Rating (VAS)			0.33 (1.41)	0.39 (1.04)	0.39 (1.42)	24.50 (18.54)	<0.001*	1.00	
Blood Markers (pg/ml)		IL-1β	4.76 (2.8)	5.03 (3.20)	4.89 (3.22)	4.55 (2.72)	0.05	−0.22	0.19
		IL-6	6.12 (7.75)	5.37 (7.33)	5.99 (7.62)	5.35 (7.26)	0.53	0.08	0.64
		IL-10	15.11 (13.87)	14.91 (13.62)	15.49 (13.57)	14.65 (13.25)	0.32	−0.09	0.59
		TNFα	8.17 (2.40)	8.27 (2.70)	8.26 (2.48)	8.08 (2.65)	0.32	−0.24	0.15
Average EEG Frequency Band Power (V^2^)	Frontal	Theta	11.05 (13.02)	12.36 (11.6)	14.04 (13.66)	15.08 (15.5)	0.96	−0.11	0.54
		Alpha	16.03 (15.30)	13.73 (15.88)	16.79 (15.72)	15.69 (15.12)	0.50	−0.10	0.56
		Beta	7.36 (4.06)	7.14 (3.91)	7.26 (4.19)	7.77 (3.73)	0.44	−0.07	0.68
		Gamma 1	5.49 (5.22)	5.45 (5.40)	5.67 (4.99)	6.75 (5.79)	0.31	−0.10	0.56
		Gamma 2	4.42 (3.98)	4.31 (3.53)	4.72 (3.33)	5.01 (3.93)	0.69	−0.04	0.82
	Central	Theta	5.29 (3.04)	6.52 (3.98)	7.24 (6.01)	7.17 (4.77)	0.45	−0.17	0.32
		Alpha	13.02 (10.68)	10.64 (10.34)	14.21 (12.86)	13.14 (10.54)	0.42	−0.09	0.59
		Beta	5.78 (3.44)	5.40 (2.90)	5.74 (3.27)	5.69 (2.87)	0.73	0.07	0.67
		Gamma 1	2.60 (2.27)	2.9 (4.93)	2.89 (2.7)	2.50 (2.27)	0.60	0.03	0.89
		Gamma 2	2.16 (2.4)	2.55 (5.92)	2.71 (3.42)	2.10 (2.38)	0.51	0.02	0.90
	Temporal	Theta	7.31 (5.63)	8.62 (6.25)	9.77 (8.33)	9.72 (8.04)	0.15	−0.15	0.39
		Alpha	17.53 (14.95)	14.09 (16.63)	19.56 (18.94)	16.88 (17.42)	0.63	−0.08	0.65
		Beta	9.63 (5.21)	8.36 (3.3)	8.10 (4.28)	8.76 (5.52)	0.22	0.21	0.22
		Gamma 1	8.09 (7.07)	6.83 (3.6)	5.79 (5.17)	6.90 (8.74)	0.41	0.20	0.25
		Gamma 2	6.03 (3.65)	6.1 (3.49)	4.85 (4.64)	5.81 (7.97)	0.66	0.13	0.46
	Parietal	Theta	6.94 (6.26)	9.58 (10.6)	8.79 (9.09)	9.70 (9.55)	0.42	−0.14	0.42
		Alpha	31.04 (32.66)	26.53 (35.1)	32.5 (35.46)	32.02 (35.78)	0.21	−0.07	0.68
		Beta	8.23 (6.40)	8.17 (5.40)	8.04 (6.46)	8.36 (5.85)	0.71	0.16	0.36
		Gamma 1	2.29 (1.10)	2.58 (1.87)	2.18 (1.84)	2.05 (1.16)	0.25	0.13	0.44
		Gamma 2	1.85 (1.36)	2.04 (1.82)	1.68 (1.81)	1.49 (1.13)	0.32	0.09	0.61
	Occipital	Theta	8.96 (7.36)	9.96 (8.35)	9.49 (6.64)	9.76 (6.55)	0.59	−0.13	0.46
		Alpha	55.48 (65.79)	50.79 (77.91)	60.62 (70.14)	57.24 (70.81)	0.83	−0.22	0.20
		Beta	12.89 (6.46)	12.5 (8.00)	13.73 (8.4)	12.67 (6.89)	0.59	−0.01	0.94
		Gamma 1	8.50 (7.70)	9.55 (12.29)	9.38 (12.17)	7.82 (8.70)	0.28	−0.02	0.91
		Gamma 2	7.02 (6.81)	8.32 (10.37)	7.94 (11.07)	6.95 (9.37)	0.26	−0.04	0.83
	Brain (all regions)	Theta	7.55 (5.53)	9.2 (6.88)	9.64 (7.73)	10.08 (7.35)	0.49	−0.16	0.35
		Alpha	23.48 (22.68)	20.14 (25.12)	25.2 (25.07)	23.82 (24.65)	0.38	−0.11	0.51
		Beta	8.08 (3.98)	7.74 (3.50)	7.86 (4.18)	8.07 (3.74)	0.44	0.12	0.49
		Gamma 1	4.53 (1.88)	4.66 (2.61)	4.36 (2.97)	4.49 (2.76)	0.95	0.09	0.60
		Gamma 2	3.63 (1.85)	3.95 (2.65)	3.69 (2.72)	3.62 (2.79)	0.80	0.05	0.76

* Indicates a statistical significant *p*-value.

**Figure 4:**
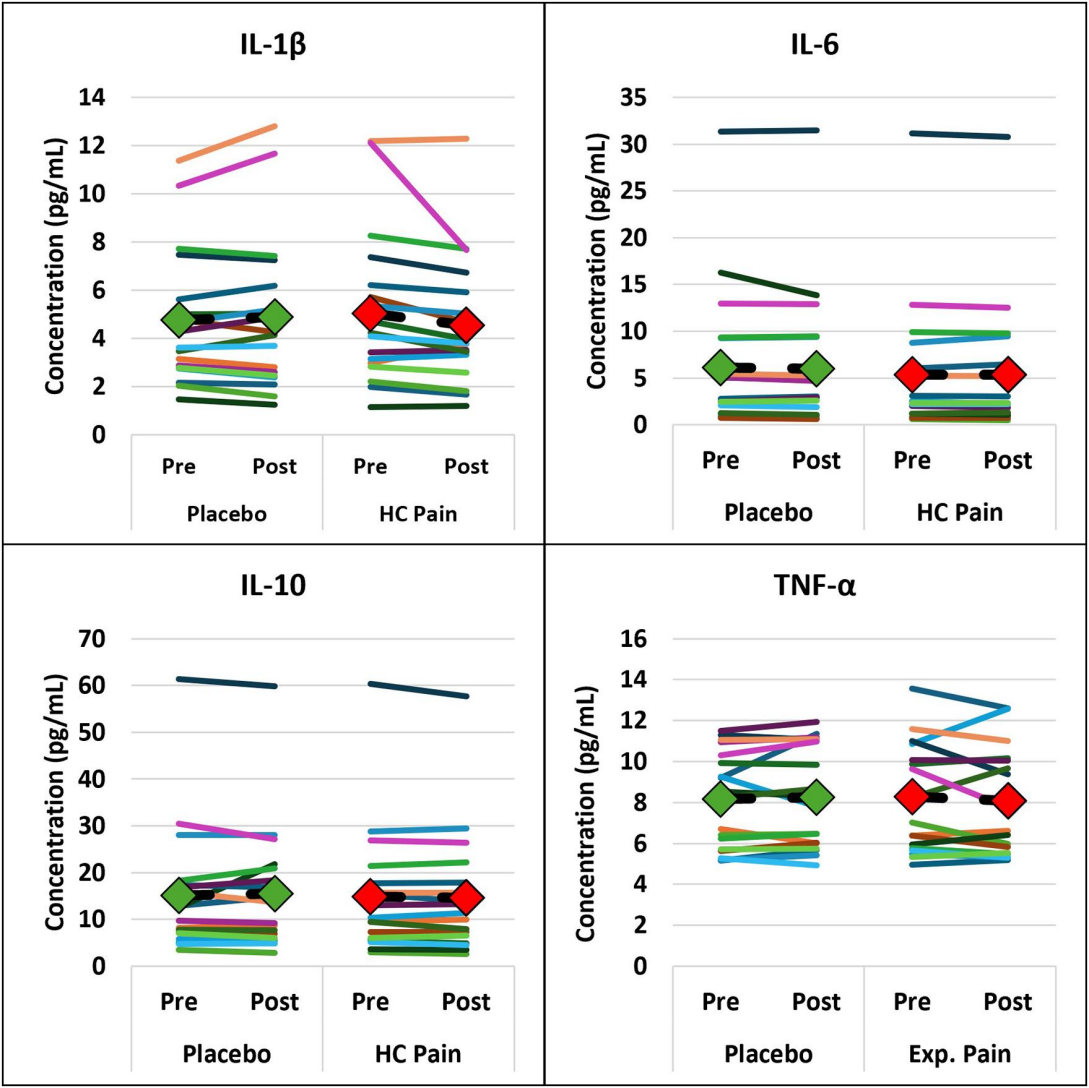
Inflammatory blood markers were not significantly different between HC pain and placebo. Individual changes from pre- to post-condition were small and occurred in both directions. The diamonds and dashed black lines represent the averages for each blood marker, with green = placebo and red = HC pain.

**Figure 5:**
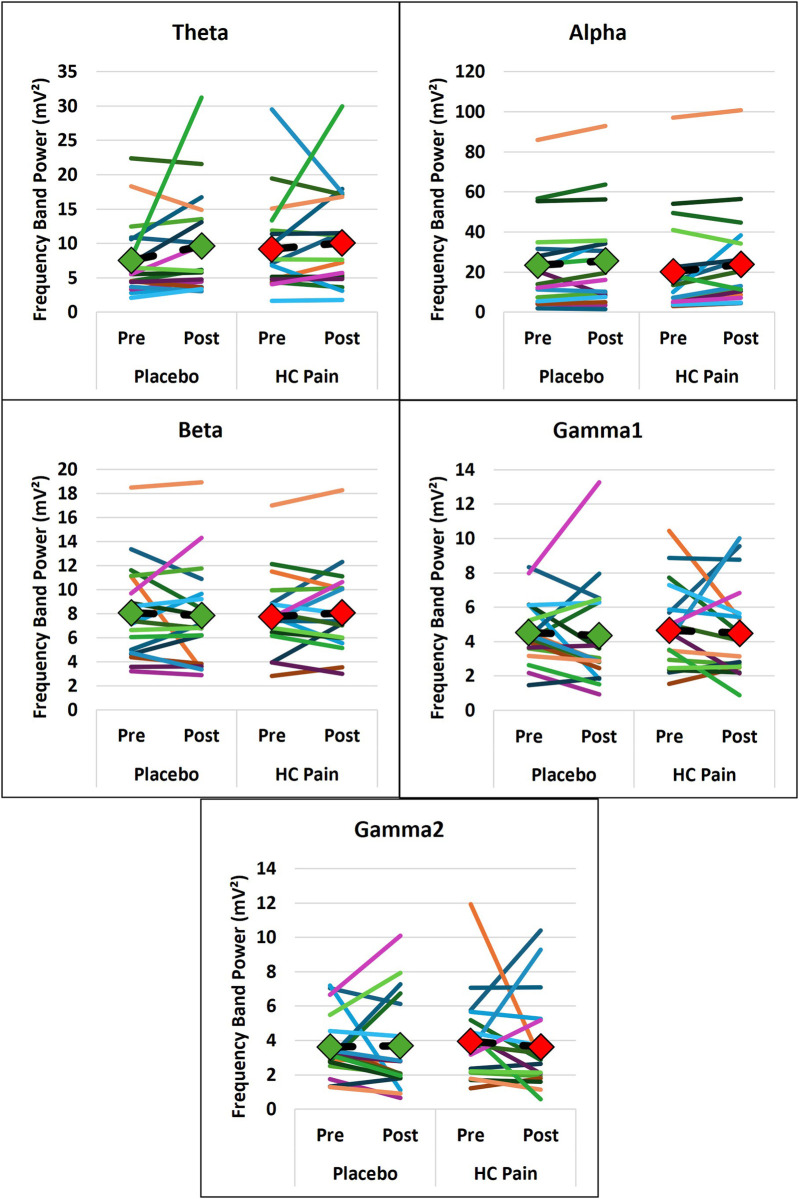
Individual changes in the EEG frequency bands show that individual changes from pre- to post-condition were small and occurred in both directions. EEG frequency bands were not significantly different between HC pain and placebo. The diamonds and dashed black lines represent the averages for each blood marker, with green = placebo and red = HC pain.

EEG and blood biomarkers are presented in Table 2. EEG and blood outcome measures remained similar at pre- and post-condition and no significant differences were found between the HC pain and placebo conditions. Figures 4, 5 show that changes of blood markers and power in the frequency bands over the global brain from pre- to post-conditions were small and variable for each individual. Similarly, no significant correlations were found between perceived pain ratings and any of the objective blood or EEG measures post-condition (Table 2).

## Discussion

This was the first study to investigate blood and brain markers of pain and inflammation in response to an experimental LBP model. The HC pain model induced mild to moderate pain over the low back in 78% of our study population and pain ratings were significantly different between experimental pain and placebo conditions. However, this perceived pain was not reflected in differences in either the EEG or blood markers between conditions. Overall, the magnitude of changes in biomarkers were small, which may have contributed to these findings. Secondarily, our study found no correlations between perceived pain ratings and post-condition measures of pain and inflammation.

Our HC model of LBP was representative of those used in recent biomechanics studies, and post-measures were completed shortly after HC application (specifically within 15 min of application of the capsaicin cream). While we were successful in inducing perceived pain similar to previous studies (3–5), this was not reflected on a neurophysiological level at the point of data collection. However, previous studies have identified that in response to the HC model, neurogenic inflammation and neuronal adaptations occur following the activation of TPRV1 receptors and C-fibers (6). Proposed adaptations include hyperactivity in supraspinal areas (21), altered descending controls (22), and changes within the dorsal horn of the spinal cord and the efferent pathways (23). Additionally, TRPV1 activation is directly linked to the release of inflammatory cytokines (22). While we also measured levels of inflammation, perhaps our null findings were due to the short duration between pain induction and data collection of the physiological outcomes.

Blood markers can provide an estimate of the level of systematic inflammation which is thought to play an integral role in the development of neuropathic pain. Pain and inflammation are linked as noxious stimuli result in the release of inflammatory cytokines which facilitate the perception of pain. Inflammatory cytokines also enter the blood stream to create a systemic response (24) but limited evidence exists for how long a pain experience must last to observe changes in systematic inflammation. Price et al. (7) assessed IL-6 and IL-10 specifically after HC pain induction over the calf. They applied the capsaicin cream followed by heat of ∼37°C for 10–15 min. While they also showed no changes in IL-10, they found increases in IL-6 concentration 30–120 min after the heat was removed compared to baseline before HC application. However, they did not measure cytokine concentrations immediately after the HC application which is when we took our blood sample. Cruz-Almeida and colleagues (2017) found an increase in pro- (IL-6, IL-8) and anti-inflammatory (IL-4, IL10) cytokines when using a cold pressor task and a focal heat pain model respectively to induce moderate pain (40–50/100 mm). However, they found that the peak changes occurred approximately 45–90 min after the pain exposure suggesting the possibility that an inflammatory response was triggered at the point of data collection but was not yet measurable in the blood. The evidence suggests that IL-6 and TNF-alpha respond immediately to exercise-induced muscle damage, stress, and inflammation while IL-1β and the anti-inflammatory IL-10 typically rise after the initial burst of inflammatory cytokines (25, 26). In addition, pre-clinical studies observed that the activation of TRPV1 channels may act to protect against systemic inflammation, thus influencing the transition from a local to a systemic inflammatory state (27). Since the HC model primarily acts through the TRPV1 channel present in the nociceptive fibres, it may have acted through a similar mechanism, inhibiting or delaying systemic inflammation measured through plasma cytokines in the present study. However, it does not appear that a systemic inflammatory response would be the driving factor for the biomechanical adaptations found. It also appears that the HC model does not replicate the increased levels of systematic inflammation found in clinical LBP patients within the time frame of data collection. However, clinical LBP typically occurs for longer periods. Future research should consider including the measurements of other markers associated with inflammation that might rise before the markers assessed in the study, such as oxidative stress markers, while also considering blood draws over longer periods of time.

Our results also showed no differences in EEG band power between the HC pain and placebo conditions. This differentiates our pain model from chronic LBP which is associated with neurophysiological changes (8). Larie et al. (8) found a widespread increase in band power in chronic LBP patients during resting EEG trials, suggesting cortical overactivity and altered pain processing. This evidence of a physiological change of neurological pathways is also observed with negative emotional states such a psychiatric disorder which often co-occur with chronic LBP (28). It is thought that pain intensity is positively associated with increases in prefrontal gamma and beta power (10, 29) demonstrating a pathway to the higher function area with the interpretation of the stimulus being dependent on contextual, integrative, and emotional factors. Phasic experimental pain on the other hand, which would be more representative of a simple noxious stimulus, has been shown to be associated with a complex spectral-temporal-spatial neuronal response starting out over the sensorimotor cortex. Longer duration, moderate pain (10 min, ∼5/10 pain score) demonstrates the shift to the prefrontal cortex: pain intensity was positively associated with gamma power (12). Our HC pain model would also be considered as longer duration pain but at a lower intensity compared to Nickel et al. (12). Thus, any short-term changes that would be similar to the phasic pain models might be washed out by the duration of time or lower intensity. Similarly, it is unclear how different stimulus/pain intensities would affect these changes. While our results showed no correlation between perceived pain ratings and brain activity and inflammatory markers, the different perceived pain ratings all occurred in response to the same noxious stimulus. Perceived pain ratings only indicated mild to moderate pain, with most pain ratings ranging between 15 and 50 out of 100 mm. Future studies should increase the noxious stimulus and/or increase the duration of the pain stimulus to determine whether the HC LBP model affects the neurological pathway similarly to the thermal heat pain model that Nickel et al. (12) applied.

Our study has a few limitations including the small sample size of 18 which is offset by the within-subject design. Additionally, our study compared the HC pain to a placebo condition and participants were blinded to minimize psychological confounders. Although the HC model has been extensively used (30–33), it has typically been applied to the forearm while we applied it to the low back. Our results showed a spread of peak perceived pain ratings for the HC model between 0 and 71. While this is representative of a clinical population, it did not create a homogeneous pain population as intended which could be achieved by monitoring pain levels and adjusting the temperature accordingly. Lastly, our pain model was representative of those used to investigate the impact of current perceived pain on biomechanical parameters. Thus, we took our outcome measures shortly after the pain/placebo induction to reduce the burden on participants. However, future studies should repeat the measures after longer periods of time to establish if any longer-term adaptations occur.

In conclusion, when HC LBP is used to study outcomes immediately after pain induction, as it is commonly done in biomechanics studies, the level of perceived pain is not an indication of EEG and blood markers within this time window. Further studies are needed to determine whether an inflammation response was triggered but not yet measurable. Similarly, more research needs to be completed to determine whether the pain exposure was intense and/or long enough to create neurophysiological changes. The HC pain model has the potential to induce perceived pain and allow for within-subject analyses, however, it should be noted that this perceived pain does not represent clinical pain on a neurophysiological level.

## Data Availability

The raw data supporting the conclusions of this article will be made available by the authors, without undue reservation.
